# Exploring the Role of Leadership in Facilitating Change to Improve Cancer Survival: An Analysis of Experiences in Seven High Income Countries in the International Cancer Benchmarking Partnership (ICBP)

**DOI:** 10.34172/ijhpm.2021.84

**Published:** 2021-08-04

**Authors:** Melanie Morris, Maureen Seguin, Susan Landon, Martin McKee, Ellen Nolte

**Affiliations:** Department of Health Services Research & Policy, London School of Hygiene & Tropical Medicine, London, UK.

**Keywords:** Cancer Outcomes, Health Systems, Leadership, Governance, High-Income Countries

## Abstract

**Background:** The differences in cancer survival across countries and over time are well recognised, with progress varying even among high-income countries with comparable health systems. Previous research has examined several possible explanations, but the role of leadership in systems providing cancer care has attracted little attention. As part of the International Cancer Benchmarking Partnership (ICBP), this study looked at diverse aspects of leadership to identify drivers of change and opportunities for improvement across seven high-income countries.

**Methods:** Key informants in 13 jurisdictions were interviewed: Australia (2 states), Canada (3 provinces), Denmark, Ireland, New Zealand, Norway and United Kingdom (4 countries). Participants represented a range of stakeholders at different tiers of the system. They were recruited through a combination of purposive and ‘snowball’ strategies and participated in semi-structured telephone interviews. Interview transcripts were analysed thematically drawing on the World Health Organization (WHO) health systems framework and previous work analysing national cancer control programmes (NCCPs).

**Results:** Several facets of leadership were perceived as important for improving outcomes. These included political leadership to initiate and maintain progress, intellectual leadership to support those engaged in local implementation of national policies and drive change, and a coherent vision from leaders at different levels of the system. Clinical leadership was also viewed as vital for translating policy into action.

**Conclusion:** Certain aspects of cancer care leadership emerged as underpinning and sustaining improvements, such as appointing a central agency, involving clinicians at every stage, ensuring strong leadership of cancer care with a consistent political mandate. Improving cancer outcomes is challenging and complex, but it is unlikely to be achieved without effective leadership, both political and clinical.

## Background

**Key Messages**

** Implications for policy makers**

- To narrow the gaps in cancer outcomes between high-income countries, attention must be focused on the leadership structures in place for making and implementing change.
- Central bodies or agencies can be pivotal for providing long-term follow through of cancer plans and strategies, and can wield funding levers to ensure implementation.
- Clinicians and leaders at every tier of the cancer care system should be involved at every stage of strategy development and implementation so that policies are relevant and likely to be implemented.
- Political will is vital to provide a strong mandate for those leading cancer care and the changes needed.

** Implications for the public**

There are persistent differences in the cancer outcomes of high-income countries which might be expected to have similar access to healthcare and technologies. Some of this difference might be due to how cancer care is organised in each country and how the cancer care system is led. We found that some features of more successful countries were: central cancer agencies, the involvement of clinicians and all levels of cancer care leadership in planning and implementing the cancer plans, and strong political will to follow through on cancer plans with funding. We believe these things could help to improve cancer outcomes in countries that may have some resource restrictions, but are generally able to provide good healthcare for their whole population. Cancer care could be more seamlessly delivered, treatments could be more equally distributed and long-term plans (for example for equipment or infrastructure updates) could be followed through more reliably.

Cancer survival has improved substantially in recent decades but continues to vary widely internationally,^1^ even among countries with seemingly similar health systems.^2,3^ Existing research has explored reasons for these differences, focusing on the continuum of care from awareness of symptoms^4-9^ and timely diagnosis^10^ to cancer treatment, aftercare and monitoring of patients.^11^ The role of health services and systems in determining outcomes is widely acknowledged, with attention to issues such as regulation and financing,^12^ primary care^13^ or availability of and access to timely diagnosis and treatment.^3,14-21^ However, a precise determination of how characteristics of health systems contribute to cancer outcomes has been difficult,^12,22-24^ likely in part due to the complex interconnections between patients, health service organisations, and health systems.^25,26^

 We previously argued for a systems approach to understand how aspects of health systems impact cancer outcomes.^26^ Leadership, and governance, now seen as a key building blocks of a health system, are crucial for achieving high performance.^27^ Conceptions of leadership vary widely, with one widely cited definition describing it as “the process of influencing others to understand and agree about what needs to be done and how to do it, and the process of facilitating individual and collective efforts to accomplish shared objectives” (p. 23).^28^ Leadership can be assumed by different actors within the health system. Although overall leadership is a government’s responsibility^29^ and necessitates a political mandate, some leadership functions in service organisation and delivery, including in cancer care, tend to be delegated to subordinate bodies such as health authorities, non-governmental agencies, or professional associations. These might be national, regional and/or local, often reflecting the constitutional arrangements in a given country, whether it is a unitary state or federation, and the distribution of responsibilities for health policy within it.^30^

 Within complex health systems, leadership is by consent, creating the conditions for success, the incentives for those who must deliver services, and holding them to account.^29,31^ Leadership should be assessed by its ability to identify where change is needed, design appropriate responses, implement them, and ensure that objectives are achieved. In the context of cancer care, experience in many countries points to the importance of having a cancer strategy setting a clear vision of what is needed and how it will be achieved, and which delivers a high level of ownership among those who must make it work.^30,32^ Political leadership is equally important, as is the intellectual and technical leadership from individuals, reflecting the complex and complicated nature of healthcare. In addition, it is essential to have clinical leadership, assembling, managing, inspiring, and evaluating the multidisciplinary teams that are necessary to deliver modern health services. All of these manifestations of leadership are interdependent. Success is unlikely if any of them are missing. Moreover, they must operate at all levels of the entire system (in primary, secondary and tertiary care) based on effective communication along the whole pathway followed by the patient on their journey from initial diagnosis through treatment to cure or palliation. There is growing evidence linking the style and quality of leadership at different tiers of a health system to health outcomes^33,34^ but in particular at the level of the hospital.^35,36^ However, little research has systematically studied the role of leadership in relation to cancer care and how this may be linked to cancer outcomes such as survival.

 This study seeks to fill this gap by exploring the role of leadership in cancer care delivery in seven high-income countries, reflecting on experiences of stakeholders within the system. It is part of the work of the second phase of the International Cancer Benchmarking Partnership (ICBP-2)^37,38^ which has brought together a wide network of policy-makers, academics, and clinicians researching cancer outcomes in a set of comparable high-income countries (Australia [3 states], Canada [10 provinces], Denmark, Ireland, New Zealand, Norway, the United Kingdom [4 nations]), all of which have high-quality population-based cancer registries, universal access to healthcare funded from general taxation, and spending comparable shares of their national income on health. The approach taken in this paper was guided by a conceptual model depicting interrelationships and causal pathways linking elements of health systems and cancer survival along the patient journey. Leadership is conceptualised as a key element all along this pathway.^26^ We explore themes identified by stakeholders in relation to the different loci of leadership within the system.

## Methods

 We used a qualitative exploratory design,^39,40^ gaining insights by gathering information from those directly involved in the topic under investigation. We draw on key informant interviews from a subset of the jurisdictions covered by ICBP-2, complemented by a review of cancer policies and strategies from each of the jurisdictions (published 1995-2018) to place interview insights in context.

###  Data Collection: Document Review

 Cancer policies and strategies were identified through an iterative process of searching through government websites and through contacts on the ICBP Programme Board. ICBP representatives from each jurisdiction were consulted to ensure completeness of the process.

###  Data Collection: Key Informant Interviews

 Key informant interviews were carried out in 13 jurisdictions between January 2019 and March 2020: New South Wales and Western Australia in Australia; Manitoba, Nova Scotia (NS) and Ontario in Canada, Denmark, Ireland, Norway, New Zealand, and the four UK nations (England, Northern Ireland, Scotland, Wales). Given the devolved nature of service organisation in Australia, Canada and the United Kingdom we also included national stakeholders to provide insights into policies and regulations national levels. The subset of jurisdiction was selected to give a broad representation of different outcomes, as well as a range of population sizes and geographical distributions.

 Interviews sought to capture a range of stakeholders directly and indirectly involved in the planning, organisation or delivery of health and/or cancer services (hospital managers, regional authorities and government officials, including of arms’ lengths bodies), along with representatives of professional bodies and of patient associations, and other relevant agencies with cancer or health policy expertise. Key informants were identified through a combination of purposive and ‘snowball’ strategies, working with local ICBP-2 collaborators and searching websites of key organisations involved in policy development or decision-making at local level delivery of cancer services.

 The range of experts interviewed varied with the population size of the jurisdiction and the way health services are organised and governed. Informed by our previous work,^41,42^ we sought to recruit approximately eight experts in Australia, 20 across Canada, 6-8 each in Denmark, Ireland, New Zealand and Norway, and approximately 20-22 across the four countries of the United Kingdom.

 Interviews were conducted by telephone or by videoconference due to geographical distances; this approach to interviewing is considered to be as effective as face-to-face interviews.^43^ The topic guide for interviews used a semi-structured format^44^; it was developed iteratively, informed by our conceptual model^26^ as well as themes that were identified from our review of cancer documents. The guide was piloted among clinicians and other members of the ICBP project board with feedback incorporated. Interviews explored stakeholders’ views on cancer policies and services in their jurisdictions; likely explanations for observed improvements and variations in cancer survival; key achievements and (continued) challenges in cancer service organisation and delivery along the patient journey; and perceived effectiveness of policy levers and instruments driving cancer services and policies. The topic guide included survival trends for four illustrative cancer sites in each of the seven countries for the period 1995-2014 (oesophagus, rectum, pancreas and ovary; Supplementary file 1), reflecting some of the eight cancers which are the focus of ICBP-2. These had different outcomes among and within countries. However, we did not limit discussion to them: health system mechanisms often operate across cancers and so any were considered during the interviews, according to participants’ expertise or interests. We also provided an illustration of the diagnostic and treatment elements of the cancer patient journey, abstracted from our conceptual model,^26^ to facilitate discussions of enablers and barriers in cancer service organisation and delivery in each jurisdiction (Figure).

**Figure:**
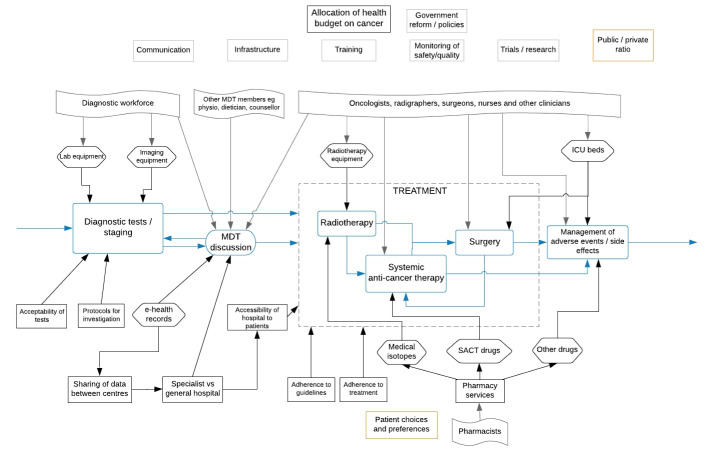
The Cancer Patient Journey: A Simplified Model. Abbreviations: SACT, systemic anti-cancer therapy; ICU, intensive care unit. Source: adapted from Morris et al.^26^

 Interview participants were provided with a project information sheet, topic guide and consent form in advance to the interview. Three authors [MM, MS, EN] conducted the interviews. The first 20 interviews were conducted by at least two authors to allow for reflective questions from the second interviewer. It also ensured that as ‘solo’ interviews started to be undertaken, there was a consistent protocol followed by all. At this point, each of the three authors took the lead on a particular jurisdiction to ensure consistency of approach. Interviews were recorded with participants’ permission, and transcribed verbatim. Two interviews were not recorded but the content was recorded in contemporaneous notes.

###  Interview Analysis 

 Analysis of interview transcripts employed a thematic approach, progressing through data familiarisation, coding, and theme refinement. Transcripts were read, cleaned and re-read by two authors [MM, MS]. The qualitative textual analysis initially followed nodes generated from the interview topic guide and informed by the World Health Organization (WHO) health systems framework^27,29^ and that developed by Atun et al for the analysis of national cancer control programmes (NCCPs),^45^ developing a coding scheme.^44^ Transcripts were coded in NVivo 12 software^46^ by individual researchers, who assigned a tentative theme (or ‘node’) to each meaningful phrase. Themes were then discussed among the research team, with nodes grouped together and re-coded into overarching themes.^39^ In this paper we focus on the themes that emerged around leadership and governance. The results included below reflect the situation and progress in each jurisdiction at the time of the interview. Jurisdictions may have developed new cancer strategies since then, eg, an updated Cancer Action plan was released in New Zealand and a new National Cancer Control Agency established after the interviews.

## Results

 We interviewed 79 people representing six categories of organisations across the 13 jurisdictions (Table). Seventy interviews were conducted (six interviews included more than one participant), lasting an average of 54 minutes (range 35 to 77 minutes).

**Table:** Organisations Represented, by Interview Participants and Country

**Jurisdiction**	**Type of Organisation (Main Role)**						
	**National/ Regional Government **	**Regional/Local Hospital Management**	**Professional Association**	**Patient Organisation**	**Academia**	**Other** ^a^	**Total**
Australia^b^	5	2	1	1	1	1	**11**
Canada^c^	8	7	2	1	1	-	**19**
Denmark	1	1	1	2	1	1	**7**
Ireland	2	1	1	-	1	1	**6**
New Zealand	3	-	1	-	1	2	**7**
Norway	1	2	-	-	1	1	**5**
UK^d^	7	6	2	3	1	5	**24**
**Total**	**27**	**19**	**8**	**7**	**7**	**11**	**79**

^a^ For example cancer societies and charities. ^b^ Including Pan-Australia, Western Australia, New South Wales. ^c^ Including Pan-Canada, Manitoba, Ontario, Nova Scotia. ^d^IncludingEngland, Northern Ireland, Scotland, Wales.

 Within the overarching themes of leadership and governance, participants raised issues that fell into four sub-themes, each identified as important potential drivers for change that would ultimately shape cancer survival: (1) political leadership steering the development of cancer services; (2) intellectual leadership by those possessing a set of technical competences and skills that can bring about change (transformational leadership), combined with a dedicated political mandate; (3) the inclusion of clinical leadership in decision-making and implementation, and (4) leadership across the different tiers of the system. We report on these in turn.

 It is important to emphasise that most participants highlighted the complexity of factors that may have contributed to observed improvements in cancer survival over time in their jurisdictions. This was especially true for leadership, where direct connections were seldom made with improvements in particular outcomes.

###  Political Leadership

 Participants reported that political engagement with cancer care often came about in response to public and media reports on their jurisdiction’s performance on cancer outcomes with others that were seen to be doing better. Study participants highlighted the role that a series of publications from the 1990s onwards, such as the EUROCARE^47^ and CONCORD studies,^48^ had played in prompting action, leading to policies that included increased investment in services and infrastructure. In some cases, the policy response led to the creation of new agencies charged with providing leadership of cancer strategies and, in some, placing individuals renowned for their cancer expertise into leadership positions. Taken together, these measures were viewed by many participants as contributing to sustained progress.

 The English 2000 Cancer Plan^49^ was one strategy that emerged following these early reports, cited by almost all English participants as having played a critical role in subsequent improvements in cancer services. Similarly, in Denmark, international benchmarking was seen to have placed cancer firmly on the national policy agenda, with a national Cancer Steering Group established in 1998 by the Ministry of Health, “based on a public debate about the quality of cancer treatment and results in Denmark compared to the countries around us” (p. 5).^50^ Its members were tasked with drawing up the first national cancer plan (published in 2000), which was accompanied by significant investment in expanded diagnostic and treatment capacity.^50^ Norway published its first national cancer plan in 1997, and again this was seen to have been stimulated by evidence of lack of progress in cancer care compared to other countries, leading to increased investment in diagnostic and treatment capacity.

 In Ireland, the political commitment that arose from international benchmarking was seen to have contributed to a debate that led to the establishment of the 2006 NCCP:

*“I think that the whole transformation of cancer services was certainly led. They looked at other countries to see what the best model for care was or are and they followed up their decisions with funding so I think politicians across all political parties accept that this was a good decision made at the time and has yield[ed] the benefits so I don’t think there’s any question in the political arena at those policy decisions…and the strategy that came out in 2006 and 2017” *(R24_Ireland).

 More recently, the experience of rapid diagnostic clinics in Denmark was seen to have prompted senior clinicians to persuade policy-makers to make changes:

*“I think [that] was the moment where some of our leading clinicians who have been chomping at the bit to say ‘we need to be so much better, we need to have senior leadership buy in for this, it isn’t just about cancer clinicians, this is about Chief Executives and Executives owning this agenda’” *(R46_Wales).

 Political leadership, even if only due to outside pressure, may have led to several initiatives, including the development of Cancer Care Ontario (CCO) and the appointment of the first National Cancer Director in England (1999), which we will return to later, but this push from political leaders was more difficult to sustain. In England we heard scepticism about whether the earlier commitment had been maintained. For example, one participant said:

*“The government will focus on the politics and push organisations as to what to do, and that gets in the public eye, but it doesn’t necessarily mean they’ll open up funding coffers” *(R65_England).

 Reflecting on austerity measures following the global financial crisis, participants from several jurisdictions suggested that commitment to investment in preserving cancer services appeared to have continued and, in the United Kingdom and Ireland, cancer services had fared better than others.

 One tentative lesson from these examples is that political leadership is important, but political engagement is difficult to sustain. Sustained success appeared to be more likely where a body existed to develop and take forward a strategy. The absence of a strategy and an implementing body in Northern Ireland was cited as a reason why it was difficult to ensure sustainable services:

*“The longer-term approach to investing in cancer services, we I think have been found on the back foot with some of our services, because of a lack of attention over a number of years and then we have struggled to catch up. And I think a strategy might have afforded us a map, you know, that we could have worked over a number of years to achieve something albeit in the longer term” *(R35_Northern Ireland).

 Similarly, participants from New Zealand reported how early progress following their first cancer plan had not continued at the previous pace, with little sustained political or financial commitment, with adverse consequences for quality of care.

###  Intellectual Leadership

 Interview participants in Ireland and England highlighted the key leadership role that identified senior officials had played, both in terms of the mandate given and their individual effectiveness. Examples cited by informants include the appointment of a National Cancer Director (‘cancer tsar’) in England who served from 1999 to 2013. The leadership of the individual was seen as being important to driving change, as was the political mandate that came with the role:

 “…*he was able to galvanise clinicians, both doctors and the nurses and other health professionals who all are involved in cancer, but also manage upwards towards the ministers as well” *(R64_England).

*“Tony Blair, when he did make his commitments to appointing a ‘national cancer tsar’ as it was called […] [showed that cancer] was a national priority” *(R64_England).

 However, successful leadership requires protected time to effect change, which was noted to have reduced over time (“and I think that is part of the overall squeeze on the NHS” (R61_England)), as was the team supporting the national leadership role, which was split into two after 2013, compromising their ability to drive change.

 A similar range of leadership skills was noted by key informants in Ireland, commenting on the recruitment, in 2007, of an Interim Director of the NCCP to oversee implementation of the 2006 strategy:

 “*He’s very clear on what he wants to achieve and will go about it very quickly, and it was basically his leadership and his belief. Really, what is being done in Ontario and in other provinces in Canada, the regionalisation of care, he saw that as a success in Canada and began to implement it in Ireland” *(R10_Ireland).

 The 2014 implementation report said: “He engaged widely with radiation, surgical and medical oncologists, GP and nursing representatives and voluntary agencies.”^51^

 This form of intellectual leadership that combines vision with skills and is supported by a strong political mandate could be seen to be a template for other countries. Individual leaders were occasionally mentioned as influential in other contexts (for example, a leader of CCO, a head of the Scottish Cancer Taskforce, a General Secretary of the Cancer Society in Norway). There were also examples of more informal layers of influence, such as the ability to bring together different perspectives or having the “ear of government and chief execs” (R77_Scotland) seen as important features.

###  Clinical Leadership

 Clinical leaders may be physicians, nurses or other healthcare professionals. Clinical leadership was identified as key to driving change in most interviews, through ensuring that strategies are evidence-based and clinically focussed and lending credibility to official plans, getting people ‘on board’ in their local areas. In England, for instance, we were told that clinical leaders help to ensure that national funding for cancer is identified and used as intended at the local level, thereby increasing the probability of a good outcome.

 Clinicians can also act as ‘translators,’ conveying needs of patients to policy-makers:

 “*So, bureaucrats don’t speak clinical language, they speak policy language, and vice versa. And, what I feel that we do a lot of with the clinical teams, is turn policy into clinical language, and how we can use the policies as enablers to make a difference, and how we’ve got to keep the clinical people with a seat at the table so that we actually get policy that’s going to make a difference to the patients” *(R60_Western Australia).

 Many cancer strategies have placed clinicians at the core of planning and implementation,^38,49,52-60^ and in all jurisdictions, clinicians were reported to sit on expert or advisory groups, communicate issues to government, advise on action and implementation, write cancer strategies, or develop guidelines:

 “…*we have a network of clinical leaders, programme leaders, both provincially and in every region of the province to help design that plan but also then to be accountable for implementing it across the province” *(R02_R03_Ontario).

*“What [they’ve] actually done in the Cancer Control Programme since its inception was to bring clinicians into the programme here to give [them] advice. To help [them] figure the right direction, the right investments so [they] would have a number of clinical leads groups where each designated cancer centre would send one member of staff to represent that hospital” *(R24_Ireland).

 Local clinical leaders are frequently held to account for achieving and maintaining regional or national targets (“we have to live up to these targets. So, it’s based in the leadership system. We know our leaders, they will come after us if we don’t live up to the demands” (R01_Denmark)), implying that clinical leaders need to be reviewing the metrics that reflect their performance. Whether or not failure to reach targets (where applied) is penalised varied between jurisdictions, ranging from “verbal” reprimand (R67_Norway) to financial penalties in England, although this was not necessarily seen as a bad thing but instead providing a case for more investment:

 “*I think one of the joys of having a target is that you then have a really good way of going back to policy-makers, funders, government and saying, “Yeah, really? Without the money?” Whereas if you have no target […] you don’t measure it, you don’t know what’s going on, so you get anecdote. And anecdote is not a good way to argue any case with government, so I think targets are a two-edged sword” *(R63_England).

 While clinical leadership was seen as important, lack of clinician buy-in can have considerable impact, too. Rationalisation of services into fewer, larger hospitals was slowed in Ireland, prompting one respondent to state:

 “…*the Consultants have had a lot of power. I don’t think they have as much power now but they’ve had things their own way a lot and they’re very highly paid and they’ve practiced with not a lot of accountability and long-term contracts and, you know, why would you change that?” *(R16_Ireland).

 Some countries provided support, and even stipends for clinical leadership roles (Ontario 2011-2015^61^) while in others the historical under-development of clinical leadership was acknowledged: “Effective implementation [of guidelines] requires clinical leaders and a coordinated program of outreach, for which there is not adequate funding at present” (p. 21, Australia, 2003^52^).

###  Leadership at Different Tiers of the System

 Many countries included in this study have established national or provincial bodies, programmes or roles from the late 1990s onwards to lead cancer policy development and oversee its implementation. In several jurisdictions, sustained leadership with political backing has been provided by a cancer agency that oversees cancer care delivery and maintains control over at least some funding. This can improve consistency in implementation of policies laid out in cancer strategies and ensure that policies are followed through.^52,53,62-64^

 An example is CCO, which was tasked in 1997 to coordinate and integrate cancer treatment services across Ontario.

 “*So, there was a bit of a shift and that was because of performance and we were noticing long wait times in the province, poor outcomes and what it resulted in was a shift in how cancer services are delivered in the province was a concerted effort to look at delivery and improve delivery. So, I would say that the role of CCO has evolved because of the need and the desire to ensure equitable and safe patient care” *(R19_Ontario).

 It has been the agency “at the centre” (R02_R03_Ontario) of organising networks of leaders, both across and within provinces, leading design of the cancer plan and for holding these networks accountable for its implementation. Its control over funding for cancer services has been cited as one reason for its success:

 “*So, not only do they have oversight of the delivery and performance measurements in management, they hold the funding levers. So, they’re able to tie funding to performance and when performance doesn’t meet standards…they’ve got that stick…to use with the regional cancer programme ” *(R18_Ontario).

 In Ireland, the NCCP, part of the Health Service Executive, is charged with providing leadership, ensur[ing] implementation of the strategy, and “set[ting] standards and guidance for the delivery of cancer care and ensure the monitoring and oversight of cancer services” (p. 13). The most recent plan also sets out “an enhanced role in the funding and commissioning of cancer services” (p. 118, Ireland, 2017-2026^65^) for the NCCP. We were told:

 “[*the NCCP] have a big element of control over the budgets for cancer services.... [The] budgetary levers are two-fold really. One is the budget [they] send to hospitals to employ staff for over the course of the year, but [they] have a much bigger degree of control over the cancer drug budget” *(R24_Ireland).

 This was also the case when the cancer programme at the Nova Scotia Health Authority (NSHA) took over from Cancer Care NS, a body which “had a responsibility to advise, but did not have the power to institute.” The cancer programme “*now has budget responsibility for all cancer in the province, outpatient cancer, how the operating rooms, and that sort of thing*” (R14_NS).

 In Western Australia, the Cancer Network is a focal point, bringing together clinicians and “service provider executives”:

*“And really to break down those barriers around individual practice or personalities, or fiefdoms etc, to say ‘no, we’re going to make a determination about what we think is safe, quality care, and we’re going to have some legitimacy about how we shape that’. And, that’s, I think, been one of the things that the network’s been really helpful for, bringing together different perspectives, but then an agreed determination about a way forward, and some degree of endorsement around that...” *(R57_R58_Western Australia).

 Many jurisdictions also have bodies that might be named ‘agencies,’ such as the Cancer Agency in Saskatchewan, Cancer Institute NSW, Cancer Research UK and Cancer Council Victoria, who have important advocacy roles, but a more limited implementation responsibility. In these cases, it is often through the development of “a network of valued relationships”^63^ that the leadership role is pursued. Thus, they have a role in coordinating the different types and levels of leadership across cancer care to ensure a consistent message and alignment of effort. Continuity offered by these non-governmental bodies can mean that the development and implementation of cancer plans is seen through to completion despite leadership changes.

 Central leadership was seen by respondents everywhere as essential to drive change and ensure consistency in policies from national through to local level. This was reported to be easier in smaller jurisdictions, such as Scotland and Western Australia:

 “*When you’re trying to introduce a new initiative, or look at what’s going on with a certain population of patients within Perth, there’s definitely less people to get around the table to influence, or to have input from, so I think we have a few less egos to deal with” *(R60_Western Australia).

 Centralisation of oversight was seen to ensure consistency of service delivery, which was argued to be the case in Manitoba, NS and Norway. In the latter, there had been fragmentation across 19 counties, but more recently adherence to cancer care pathways has been monitored centrally and “*the political control is extremely strong*” (R67_Norway). We also heard similar arguments from respondents in NS where the system had changed from nine independent district health authorities into a single health authority, the NSHA; Cancer Care NS was moved into NSHA.

 A failure to assign a central leadership role has proved problematic, hindering progress in cancer care. Key informants from New Zealand highlighted the challenges posed by the system’s fragmentation of cancer services across 20 district health boards (“20 different health systems effectively,” R29_New Zealand). The 2003 cancer control strategy was widely seen as a means to overcome this fragmentation (“*actually a stunning strategy, really comprehensive, really well considered, and we saw a number of exciting things like regional cancer networks pop up which was the implementation.*” R31_New Zealand) but it did not lead to sustained action. The 2005-2010 cancer plan in New Zealand^66^ did lay out the establishment of “a Cancer Control Collaborative which will foster the collaboration at a national level of various groups and activities involved in cancer control” (p. iii) but this does not yet seem to have affected implementation of policies on the ground.

 The fragmented structures created by multiple health boards in Scotland has also been perceived as creating challenges. Participants reported that three large cancer centres “*are not linked by any governance or any accountability or any financial strings*” (R72_Scotland) which made it difficult to implement national changes, and that “*things just start getting done in different ways in different health boards*” (R66_Scotland). We were also told that there is “*a reluctance amongst health boards to give up some of their authority*” (R66_Scotland) which was viewed to be needed to reduce variation in how systems are implemented across the country.

## Discussion

 This study suggests that strong leadership, at a variety of levels of the cancer care system, is perceived as important for improving cancer outcomes, as is political leadership that enables progress on a cancer strategy to be sustained. This often relies on giving a mandate to central bodies and/or individual leaders who have oversight of and authority in the system, but it also needs the expertise and influence of clinicians, advising on strategy and making changes on the ground. These various facets of leadership should, when effective, lead inevitably to quality improvement^35,36,67^ and therefore improved outcomes for patients.

###  Political Leadership 

 Our findings suggest that political leaders often responded to impetus from external sources such as international benchmarking studies and pressure from clinicians. Strong political leadership can impact cancer outcomes through the development and implementation of a national (or provincial) strategy,^31,30^ one that has been developed by policy-makers and experts (clinicians) within the system, that is regularly revisited and updated, that steers cancer care and remains focussed on improved outcomes, but that also takes account of local pressures and allows local leaders to adapt to the local context. Cancer plans have been published in high income countries for decades and have been credited with transforming standards of care in many places. The issue of leadership only appeared explicitly in a few of the ‘earlier’ plans we studied,^52,56,59,66,68^ but has become more commonly integrated in recent times.^57,58,61,65,69-74^ The essential characteristic of leadership is that it “creates a clarity and unity of purpose, encourages team building, ample participation, ownership of the process, continuous learning, and mutual recognition of efforts made.”^32^ Here the central role of sustained political leadership is clear.

 The increasing incidence of cancer in our aging populations^75^ means that cancer care will likely remain high on the political agenda for some time,^76^ but this is in a world of increasing competition for scarce resources and complexity of treatments that are already beginning to stretch expertise, capacity and infrastructure. This may be particularly relevant in under-served populations, such as First Nations peoples in Canada and the Maōri population of New Zealand, where leadership including those groups will be important in addressing inequity. Stability of “a core of the leadership team” at least is key^77^ so that implementation of strategies and their promised resources can be assured over time. Commitment to sustained and predictable investment from government to support the capacity needed for the growing cancer population is vital;^78^ without this, the best leadership is still likely to founder in its goals.

 As the key informant interviews did not set out to analyse governance arrangements, it was not possible to assess more fully the influence of governance structures, or the locus of decision-making in each jurisdiction, although these will have an impact. This study clearly highlights, however, the importance of both leadership and governance in cancer services and future work should examine these additional issues in greater depth.

###  Intellectual and Clinical Leadership

 The development of cancer strategies involved clinicians to some extent in every jurisdiction. They were involved at various different tiers in decisions about the organisation of cancer care, and they were seen as important levers for change.^76^ Research into quality improvement mechanisms has shown that, at a local level, clinical involvement in implementation is enhanced by clinical leadership and that the involvement of ‘physician leaders’ in developing and implementing strategies is key.^67^ The culture of organisations,^36^ support from senior management (which would be politicians in this context), and performance monitoring (via benchmarking and targets)^35^ have all been found to be important to improving outcomes.

 Leadership also needs to be “visionary and strategic,”^79-83^ to have an overview of the long-term goals,^84^ the needs of the system and potential challenges ahead. This can be difficult in times of system reorganisation and when scarcity of resources mean that leaders do not have the time to devote to strategic planning. Individuals involved ‘on the ground’ can play an important role here: “expertise-driven practice” as Taylor et al call it, is vital^35^ and “clinician leadership is seen as being essential to make healthcare work” (p. 12).^85^

 Strong individual leaders were seen to be important, but it should be remembered that they all operated in a context of political commitment that was supportive of their work: an individual in isolation is unlikely to have achieved similar results. In addition, the system must also be “nimble” enough (R5_Ontario) to not rely on single leaders. The WHO, in their guidelines for NCCPs, asserts that a programme coordinator should have a wide range of technical skills and political influence, as well as a plethora of management, communication and personal skills.^32^

###  Leadership at Different Tiers

 Effective cancer care relies on leadership at multiple levels^30^ to be organised, implemented and delivered. Priorities operate at different levels: the national agenda dictates what should happen but this cannot always be translated into action at local level because of competing priorities. Strong cancer leadership that operates at the different tiers of the system (national, regional, local) to drive change and improve outcomes is therefore important.^84^ Leadership at the local level is key,^85^ allowing a vision that has been adopted at national level to be implemented locally, with all adhering to the same goals, and encouraging the integration of primary, secondary and tertiary care that is so necessary for smooth flow through the patient pathway.^77^ Fragmentation of a system where each health board or area makes their own decisions was seen as a clear impediment to this coherence of vision and implementation of change, as was the frequent or large reorganisation of services that led to discontinuity in leadership structures.

 Leaders need to ensure transparency and hold other system actors accountable.^29^ This implies the “management of relationships between various stakeholders in health”^31^ something that we heard cancer agencies can play a large role in. Cancer agencies can offer “transformational leadership [which] can bring key stakeholders together with the aim of improving the cancer system as a whole” (p. 7).^30^ The ability to build coalitions and effective communication between stakeholders is vital here, and this was also cited as a characteristic of effective individuals.^76^

###  Strengths and Limitations

 This study drew on interviews from a large number of key informants representing a wide range of stakeholders in the cancer care system. To maintain representation across countries, only a limited number of informants in each jurisdiction could be interviewed. We had a broad approach to recruitment, using initial suggestions from the ICBP project board, although avoiding interviewing people directly involved in ICBP projects. We tried to minimise any bias from only using personal recommendations by also using internet searches to identify people in relevant roles. However, we heard a pattern of similar responses from a variety of participants in each jurisdiction, regardless of their position, including representatives from patient organisations and charities, suggesting that our findings were not particularly influenced by recruitment method.

 Many informants held senior positions in their organisations, thus affording us a broad and deep overview of the cancer care system in their jurisdiction. However, we are aware that these were perspectives of individuals and we cannot assume that our study captured all possible views. In addition, our approach to data collection, based around a necessarily brief topic guide, means that certain themes may have been reported more in some jurisdictions than others if we did not specifically ask about it. However, the issue under consideration was apparently seen to be of crucial relevance among those participating in the study from that jurisdiction, as some themes emerged despite not being specifically elicited from the guide.

 We also acknowledge that there are many definitions of ‘leadership,’^28^ often in combination with, stewardship^86^ and governance.^27,29,87,88^ We have laid out how we have understood leadership but accept that it may be understood differently by different audiences, influencing how the notion of leadership is conceptualised in strategy documents and by study participants.

 Finally, is important to acknowledge that, ultimately, it is not possible to causally link a particular form of leadership and organisational structure directly to survival (and other cancer outcomes) given the range of elements in the system that are working together. Each health system is characterised by a particular set of relationships between the different professionals and institutions that deliver care, and it cannot be assumed that a particular strategy or service model developed in one setting can be transplanted to another, because of the particulars of the local context that are so crucial in determining of ‘what works.’ There are several components that seem necessary to improve outcomes but removing one or another will not impact in a predictable way; it is therefore difficult to say which component is more important than another one. In a similar way, we have only been able to suggest, without being definitive, patterns of approaches to what might drive change and facilitate improvements in outcomes.

## Conclusion

 Our study points to several components that appear to be conducive to achieving consistent progress in cancer outcomes. Involvement of clinicians at every stage of strategy development appears to be core for the successful implementation of policies, including involvement in developing or defining quality measures and performance targets. Presence of a lead agency or body at central or regional level, with an explicit political and financial mandate to lead the whole system, and tasked with ensuring a transparent and coherent approach to cancer care, is likely to lead to more sustained progress in policy implementation at each tier of the system. Similarly, strong leadership of cancer care at a local level, with a mandate to make changes and implement agreed policies, can ensure implementation of sometimes aspirational or intangible aims of national cancer strategies.

 Of crucial importance will be the involvement of the community being served: many cancer strategies are developed with the input of patients and the public, but it is unclear to what degree they can influence the direction or are involved in the implementation of the aims. This is an aspect we did not explore in this study, but which should be looked at in future.

## Acknowledgements

 The authors would like to thank Lucie Hooper, Samantha Harrison, Charles Norell, Shanta Keshwala and Charlotte Lynch of Cancer Research UK for managing the programme. The ICBP Clinical Committees for their advice. The ICBP Academic Reference Group for providing independent peer review and advice, in particular: Nancy L. Keating, MD, MPH. Harvard Medical School; Professor Jane Young. The University of Sydney; Dr. Stuart Peacock. Co-Director of the Canadian Centre for Applied Research in Cancer Control and Head of Cancer Control Research at BC Cancer; Professor Kathy Pritchard-Jones. University College London.

 Finally, we are very grateful to the ICBP Programme Board for their oversight and direction.

## Ethical issues

 Observational/Interventions Research Ethics Committee at the London School of Hygiene & Tropical Medicine (LSHTM Ethics Ref: 15169).

## Competing interests

 Authors declare that they have no competing interests.

## Authors’ contributions

 MM drafted the initial manuscript with help from EN. MS, SL, EN, and MMcK reviewed the draft and contributed to editing. SL, MS, MM, and EN were involved in data collection and analysis. EN and MMcK conceptualised the overall study. EN is guarantor.

## Funding

 The ICBP is funded by the Canadian Partnership Against Cancer; Cancer Council Victoria; Cancer Institute New South Wales; Cancer Research UK; Danish Cancer Society; National Cancer Registry Ireland; The Cancer Society of New Zealand; NHS England; Norwegian Cancer Society; Public Health Agency Northern Ireland on behalf of the Northern Ireland Cancer Registry; DG Health and Social Care, Scottish Government; Western Australia Department of Health; Public Health Wales NHS Trust.

## Supplementary files

Supplementary file 1. Trends in Survival From Selected Cancers, 2000-2014.

## References

[1] Allemani C, Matsuda T, Di Carlo V (2018). Global surveillance of trends in cancer survival 2000-14 (CONCORD-3): analysis of individual records for 37 513 025 patients diagnosed with one of 18 cancers from 322 population-based registries in 71 countries. Lancet.

[2] Arnold M, Rutherford MJ, Bardot A (2019). Progress in cancer survival, mortality, and incidence in seven high-income countries 1995-2014 (ICBP SURVMARK-2): a population-based study. Lancet Oncol.

[3] Walters S, Benitez-Majano S, Muller P (2015). Is England closing the international gap in cancer survival?. Br J Cancer.

[4] Forbes LJ, Simon AE, Warburton F (2013). Differences in cancer awareness and beliefs between Australia, Canada, Denmark, Norway, Sweden and the UK (the International Cancer Benchmarking Partnership): do they contribute to differences in cancer survival?. Br J Cancer.

[5] Pedersen AF, Forbes L, Brain K (2018). Negative cancer beliefs, recognition of cancer symptoms and anticipated time to help-seeking: an International Cancer Benchmarking Partnership (ICBP) study. BMC Cancer.

[6] Evans RE, Morris M, Sekhon M (2014). Increasing awareness of gynaecological cancer symptoms: a GP perspective. Br J Gen Pract.

[7] Hall N, Birt L, Banks J (2015). Symptom appraisal and healthcare-seeking for symptoms suggestive of colorectal cancer: a qualitative study. BMJ Open.

[8] Niksic M, Rachet B, Warburton FG, Forbes LJ (2016). Ethnic differences in cancer symptom awareness and barriers to seeking medical help in England. Br J Cancer.

[9] O’Mahony M, Comber H, Fitzgerald T (2017). Interventions for raising breast cancer awareness in women. Cochrane Database Syst Rev.

[10] Neal RD, Tharmanathan P, France B (2015). Is increased time to diagnosis and treatment in symptomatic cancer associated with poorer outcomes? systematic review. Br J Cancer.

[11] Winpenny E, Miani C, Pitchforth E, et al. Health Services and Delivery Research. Outpatient Services and Primary Care: Scoping Review, Substudies and International Comparisons. Southampton, UK: NIHR Journals Library; 2016. 10.3310/hsdr04150. 27195361

[12] Uyl-de Groot CA, de Vries EGE, Verweij J, Sullivan R (2014). Dispelling the myths around cancer care delivery: it’s not all about costs. J Cancer Policy.

[13] Rubin G, Berendsen A, Crawford SM (2015). The expanding role of primary care in cancer control. Lancet Oncol.

[14] Brown S, Castelli M, Hunter DJ (2014). How might healthcare systems influence speed of cancer diagnosis: a narrative review. Soc Sci Med.

[15] de Azambuja E, Ameye L, Paesmans M, Zielinski CC, Piccart-Gebhart M, Preusser M (2014). The landscape of medical oncology in Europe by 2020. Ann Oncol.

[16] Mathew A (2018). Global survey of clinical oncology workforce. J Glob Oncol.

[17] The Royal College of Radiologists (RCR). Clinical Oncology UK Workforce Census 2018 Report. London: RCR; 2019.

[18] Cherny N, Sullivan R, Torode J, Saar M, Eniu A (2016). ESMO European Consortium Study on the availability, out-of-pocket costs and accessibility of antineoplastic medicines in Europe. Ann Oncol.

[19] Davis C, Naci H, Gurpinar E, Poplavska E, Pinto A, Aggarwal A (2017). Availability of evidence of benefits on overall survival and quality of life of cancer drugs approved by European Medicines Agency: retrospective cohort study of drug approvals 2009-13. BMJ.

[20] Salas-Vega S, Mossialos E (2016). Cancer drugs provide positive value in nine countries, but the United States lags in health gains per dollar spent. Health Aff (Millwood).

[21] Coleman MP, Forman D, Bryant H (2011). Cancer survival in Australia, Canada, Denmark, Norway, Sweden, and the UK, 1995-2007 (the International Cancer Benchmarking Partnership): an analysis of population-based cancer registry data. Lancet.

[22] Ades F, Senterre C, de Azambuja E (2013). Discrepancies in cancer incidence and mortality and its relationship to health expenditure in the 27 European Union member states. Ann Oncol.

[23] Philipson T, Eber M, Lakdawalla DN, Corral M, Conti R, Goldman DP (2012). An analysis of whether higher health care spending in the United States versus Europe is ‘worth it’ in the case of cancer. Health Aff (Millwood).

[24] Stevens W, Philipson TJ, Khan ZM, MacEwan JP, Linthicum MT, Goldman DP (2015). Cancer mortality reductions were greatest among countries where cancer care spending rose the most, 1995-2007. Health Aff (Millwood).

[25] Weller D, Vedsted P, Anandan C (2016). An investigation of routes to cancer diagnosis in 10 international jurisdictions, as part of the International Cancer Benchmarking Partnership: survey development and implementation. BMJ Open.

[26] Morris M, Landon S, Reguilon I, Butler J, McKee M, Nolte E (2020). Understanding the link between health systems and cancer survival: a novel methodological approach using a system-level conceptual model. J Cancer Policy.

[27] World Health Organization (WHO). The World Health Report 2000: Health Systems: Improving Performance. Geneva: WHO; 2000.

[28] Yukl GA. Leadership in Organizations. 8th ed. Pearson; 2013.

[29] World Health Organization (WHO). Everybody’s Business--Strengthening Health Systems to Improve Health Outcomes: WHO’s Framework for Action. Geneva: WHO; 2007.

[30] French J, Sutcliffe SB (2018). Planning for cancer control programs: leadership considerations. Healthc Manage Forum.

[31] World Health Organization (WHO). Monitoring the Building Blocks of Health Systems: A Handbook of Indicators and Their Measurement Strategies. Geneva: WHO; 2010.

[32] World Health Organization (WHO). National Cancer Control Programmes: Policies and Managerial Guidelines. 2nd ed. Geneva: WHO; 2002.

[33] Weintraub P, McKee M (2019). Leadership for innovation in healthcare: an exploration. Int J Health Policy Manag.

[34] Shubeck SP, Kanters AE, Dimick JB (2019). Surgeon leadership style and risk-adjusted patient outcomes. Surg Endosc.

[35] Taylor N, Clay-Williams R, Hogden E, Braithwaite J, Groene O (2015). High performing hospitals: a qualitative systematic review of associated factors and practical strategies for improvement. BMC Health Serv Res.

[36] Braithwaite J, Herkes J, Ludlow K, Lamprell G, Testa L (2016). Association between organisational and workplace cultures, and patient outcomes: systematic review protocol. BMJ Open.

[37] Butler J, Foot C, Bomb M (2013). The International Cancer Benchmarking Partnership: an international collaboration to inform cancer policy in Australia, Canada, Denmark, Norway, Sweden and the United Kingdom. Health Policy.

[38] Independent Cancer Taskforce. Achieving World-Class Cancer Outcomes: A Strategy for England 2015-2020. London: NHS England; 2015.

[39] Bradshaw C, Atkinson S, Doody O (2017). Employing a qualitative description approach in health care research. Glob Qual Nurs Res.

[40] Neergaard MA, Olesen F, Andersen RS, Sondergaard J (2009). Qualitative description - the poor cousin of health research?. BMC Med Res Methodol.

[41] Knai C, Nolte E, Brunn M (2013). Reported barriers to evaluation in chronic care: experiences in six European countries. Health Policy.

[42] Pitchforth E, Nolte E, Corbett J (2017). Community hospitals and their services in the NHS: identifying transferable learning from international developments–scoping review, systematic review, country reports and case studies. Health Serv Deliv Res.

[43] Novick G (2008). Is there a bias against telephone interviews in qualitative research?. Res Nurs Health.

[44] Green J, Thorogood N. Qualitative Methods for Health Research. 4th ed. London: SAGE Publications Ltd (UK); 2018.

[45] Atun R, Ogawa T, Martin-Moreno JM. Analysis of National Cancer Control Programmes in Europe. London: Imperial College London; 2009.

[46] NVivo Qualitative Data Analysis Software [computer program]. https://qsrinternational.com/nvivo/nvivo-products/1999.

[47] Coleman MP, Gatta G, Verdecchia A (2003). EUROCARE-3 summary: cancer survival in Europe at the end of the 20th century. Ann Oncol.

[48] Coleman MP, Quaresma M, Berrino F (2008). Cancer survival in five continents: a worldwide population-based study (CONCORD). Lancet Oncol.

[49] Department of Health. The NHS Cancer Plan: A Plan for Investment, A Plan for Reform. England: NHS; 2000.

[50] Danish Health Authority - Sundhedsstyrelsen. National kræftplan: Status og forslag til initiativer i relation til kræftbehandlingen / National Cancer Plan: Status and proposals for initiatives related to cancer treatment. 2000.

[51] National Cancer Control Programme. Report on the Implementation of ‘A Strategy for Cancer Control in Ireland 2006’. Dublin: Health Service Executive; 2014.

[52] Clinical Oncological Society of Australia, The Cancer Council Australia, National Cancer Control Initiative. Optimising Cancer Care in Australia. Melbourne: National Cancer Control Initiative; 2003.

[53] Cancer Institute NSW. NSW Cancer Plan 2011-2015. Sydney: Cancer Institute NSW; 2010.

[54] Department of Health and Children. Cancer Services in Ireland: A National Strategy. Dublin: Department of Health and Children; 1996.

[55] National Cancer Forum. A Strategy for Cancer Control in Ireland. Dublin: Health Service Executive Cancer Services; 2006.

[56] Cancer Care Ontario. Ontario Cancer Plan 2005-2008. Ontario: Cancer Care Ontario; 2004.

[57] Cancer Care Ontario. Ontario Cancer Plan 2008-2011. Ontario: Cancer Care Ontario; 2007.

[58] Cancer Care Ontario. Ontario Cancer Plan IV 2015-2019. Ontario: Cancer Care Ontario; 2014.

[59] Department of Health. Cancer Reform Strategy. England: NHS; 2007.

[60] Department of Health. Improving Outcomes: A Strategy for Cancer. England: NHS; 2011.

[61] Cancer Care Ontario. Ontario Cancer Plan 2011-2015. Ontario: Cancer Care Ontario; 2010.

[62] Victoria State Government. Victoria’s Cancer Action Plan 2008-2011. Melbourne: Victorian Government Department of Human Services; 2008.

[63] Cancer Australia. Strategic Plan 2011-2014. Australian Government; 2011.

[64] Saskatchewan Cancer Agency. Strategic Plan 2011-2014: Beyond the Horizon in Healthcare. Saskatchewan: Saskatchewan Cancer Agency; 2011.

[65] An Roinn Slainte/Department of Health. National Cancer Strategy 2017-2026. Dublin: Healthy Ireland, Department of Health, National Patient Safety Office; 2017.

[66] Cancer Control Taskforce. The New Zealand Cancer Control Strategy: Action Plan 2005-2010. Wellington: Ministry of Health; 2005.

[67] Weiner BJ, Shortell SM, Alexander J (1997). Promoting clinical involvement in hospital quality improvement efforts: the effects of top management, board, and physician leadership. Health Serv Res.

[68] Canadian Strategy for Cancer Control. Canadian Strategy for Cancer Control: A Cancer Plan for Canada. Canadian Strategy for Cancer Control; 2006.

[69] Canadian Partnership Against Cancer. The Canadian Strategy for Cancer Control: 2017-2022. Canadian Partnership Against Cancer; 2016.

[70] Government of Alberta. Changing Our Future: Alberta’s Cancer Plan to 2030. Government of Alberta; 2013.

[71] Danish Health Authority - Sundhedsstyrelsen. Strengthened efforts in the cancer field - a healthcare presentation. 2010.

[72] Saskatchewan Cancer Agency. Strategic Plan 2015-2020. Saskatchewan: Saskatchewan Cancer Agency; 2015.

[73] Department of Health & Human Services. Victorian Cancer Plan 2016-2020: State Government of Victoria. 2016.

[74] NSW Government. NSW Cancer Plan: 2011-2015. NSW Government; 2010.

[75] Bray F, Ferlay J, Soerjomataram I, Siegel RL, Torre LA, Jemal A (2018). Global cancer statistics 2018: GLOBOCAN estimates of incidence and mortality worldwide for 36 cancers in 185 countries. CA Cancer J Clin.

[76] Bergin RJ, Emery J, Bollard R, White V (2019). Research evidence supports cancer policymaking but is insufficient for change: findings of key informant interviews from five countries. Health Policy.

[77] Timmins N. The Practice of System Leadership: Being Comfortable with Chaos. London: King’s Fund; 2015.

[78] Seguin M, Morris M, McKee M, Nolte E. “There’s not enough bodies to do the demand:” an exploration of key stakeholder views on the role of health service capacity in shaping cancer outcomes in 7 International Cancer Benchmarking Partnership countries. Int J Health Policy Manag. 2020. 10.34172/ijhpm.2020.254. PMC9808162 33589567

[79] Institute for the Future of Oncology. Oncology Leadership: Looking to the Future in a Shifting Healthcare Environment. Rockville, MD: Association of Community Cancer Centers; 2014.

[80] Caridi-Zahavi O, Carmeli A, Arazy O (2016). The influence of CEOs’ visionary innovation leadership on the performance of high-technology ventures: the mediating roles of connectivity and knowledge integration. J Prod Innov Manage.

[81] Mintzberg H. Managing. Berrett-Koehler Publishers; 2011.

[82] Wisdom JP, Chor KH, Hoagwood KE, Horwitz SM (2014). Innovation adoption: a review of theories and constructs. Adm Policy Ment Health.

[83] Bucciarelli L (2015). A review of innovation and change management: stage model and power influences. Univers J Manag.

[84] West MA, Borrill CS, Dawson JF, Brodbeck F, Shapiro DA, Haward B (2003). Leadership clarity and team innovation in health care. Leadersh Q.

[85] Committee on the Future of Healthcare. The Cancer Strategy as a Case Study of Health Service Reform: Professor Tom Keane. Ireland: Irish Parliament; 2016.

[86] Murray CJ, Frenk J (2000). A framework for assessing the performance of health systems. Bull World Health Organ.

[87] Greer SL, Wismar M, Figueras J, McKee C. Governance: a framework. In: Greer SL, Wismar M, Figueras J, eds. Strengthening Health System Governance: Better Policies, Stronger Performance. New York: McGraw Hill Education, Open University Press; 2016.

[88] Smith PC, Anell A, Busse R (2012). Leadership and governance in seven developed health systems. Health Policy.

