# Supplementary material for: Exploring the Role of Leadership in Facilitating Change to Improve Cancer Survival: An Analysis of Experiences in Seven High Income Countries in the International Cancer Benchmarking Partnership (ICBP)
Source: Int J Health Policy Manag. 2021 Aug 4;11(9):1756–66. doi: 10.34172/ijhpm.2021.84 (PMC9808244; doi:10.34172/ijhpm.2021.84)
Supplement: Supplementary file 1 — Trends in Survival From Selected Cancers, 2000-2014. [file ijhpm-11-1756-s001.pdf]

Article title: Exploring the Role of Leadership in Facilitating Change to Improve Cancer Survival: An Analysis of Experiences in Seven High Income Countries in the International Cancer Benchmarking Partnership (ICBP)

Journal name: International Journal of Health Policy and Management (IJHPM)

Authors' information: Melanie Morris*, Maureen Seguin, Susan Landon, Martin McKee, Ellen Nolte

Department of Health Services Research & Policy, London School of Hygiene & Tropical Medicine, London, UK.

(*Corresponding authors: melanie.morris@lshtm.ac.uk)

Supplementary file 1. Trends in Survival From Selected Cancers, 2000-2014.

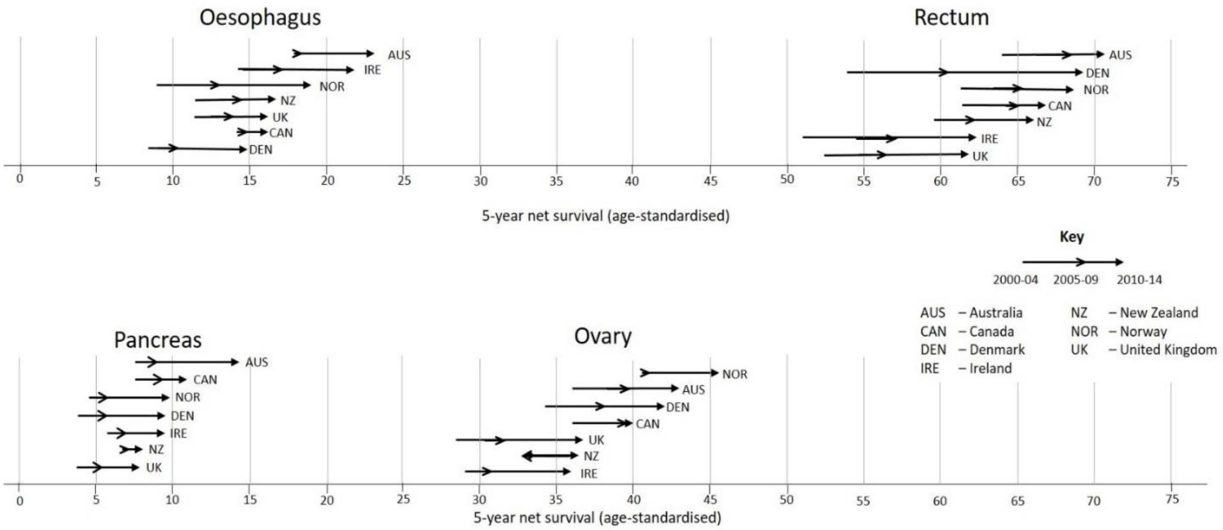

Source: adapted from Arnold et al, 2019[2]
